# The effect of simulator fidelity on procedure skill training: a literature review

**DOI:** 10.5116/ijme.5ea6.ae73

**Published:** 2020-05-18

**Authors:** Alan Kawarai Lefor, Kanako Harada, Hiroshi Kawahira, Mamoru Mitsuishi

**Affiliations:** 1 Department of Bioengineering, School of Engineering, The University of Tokyo, Tokyo, Japan; 2 Jichi Medical Simulation Center, Jichi Medical University, Tochigi, Japan

**Keywords:** Simulation education, low-fidelity, high-fidelity, laparoscopic surgery, skill assessment

## Abstract

**Objectives:**

To evaluate the effect of simulator fidelity on procedure skill training through a review of existing studies.

**Methods:**

MEDLINE, OVID and EMBASE databases were searched between January 1990 and January 2019. Search terms included “simulator fidelity and comparison” and "low fidelity" and "high fidelity" and “comparison” and “simulator”. Author classification of low- and high-fidelity was used for non-laparoscopic procedures. Laparoscopic simulators are classified using a proposed schema. All included studies used a randomized methodology with two or more groups and were written in English. Data was abstracted to a standard data sheet and critically appraised from 17 eligible full papers.

**Results:**

Of 17 studies, eight were for laparoscopic and nine for other skill training. Studies employed evaluation methodologies, including subjective and objective measures. The evaluation was conducted once in 13/17 studies and before-after in 4/17. Didactic training only or control groups were used in 5/17 studies, while 10/17 studies included two groups only. Skill acquisition and simulator fidelity were different for the level of training in 1/17 studies. Simulation training was followed by clinical evaluation or a live animal evaluation in 3/17 studies. Low-fidelity training was not inferior to training with a high-fidelity simulator in 15/17 studies.

**Conclusions:**

Procedure skill after training with low fidelity simulators was not inferior to skill after training with high fidelity simulators in 15/17 studies. Some data suggest that the effectiveness of different fidelity simulators depends on the level of training of participants and requires further study.

## Introduction

The former paradigm for teaching medical procedures, “see one, do one, teach one”, has no basis in educational theory and has been abandoned. Simulation education in medicine is being revised in an educational theory framework. Deliberate practice, cognitive task analysis and proficiency-based training are being used to design improved educational programs.^1^ Laparoscopic surgery training in the late 1980s and early 1990s often proceeded without verification of skills or competency.^2^ Since that time, training to perform procedures has undergone widespread change and simulation has become an important component. Selecting the optimal simulator for a specific procedure and trainee group is difficult.

Simulator fidelity is must be considered when developing simulation curricula. The definition of fidelity is a crucial issue and not consistently applied.^3^^,^^4^ There are both objective (mathematical) and subjective definitions (based on a trainee’s performance matrix) of fidelity.^5^ Some authors have differentiated between psychological fidelity and engineering fidelity.^6^ While some feel that fidelity relates to the replication of reality, a recent study suggests that an accurate representation of cues and stimuli is more important.^7^ There is no standard definition of fidelity for laparoscopic simulation and no accepted classification.

Having established the importance of simulation education and the wide range of definitions of fidelity, it must be determined whether fidelity is related to performance. The relationship of simulator fidelity and educational outcomes has been evaluated for over 50 years with conflicting results regarding the connection between learning and fidelity.^4^^,^^8^^,^^9^  Higher fidelity may not translate into more effective training, and lower fidelity simulation may improve training and education.^9^  It assumed that the closer a simulator is to the “real world”, the better the transfer of skills to clinical care.^6^ It is also assumed that more complex skills require more complex simulators.^6^ These assumptions lead to the conclusion that skill transfer with high fidelity (HF) simulators is better than with low fidelity (LF) simulators.^10^ Despite the assumptions which may appear to be self-evident, there is little evidence to support them, particularly in regard to procedure skills training.^6^^,^^9^

There are few reviews of fidelity directly comparing HF and LF simulation in medical education.^6^^,^^11^^,^^12^ There is a review of only HF simulators^13^ and another review of only LF simulators.^14^ The lack of a standard definition or classification of simulator fidelity complicates such studies. Norman and colleagues performed a focused review of 24 studies comparing LF and HF simulators for examination skills, procedural skills and scenario management.^6^ They included seven studies of procedural skill training, specifically reviewing the association of fidelity and skills transfer and concluded that there is no association. Nguyen and colleagues conducted the only systematic review to date that specifically evaluates simulator fidelity for laparoscopic skills, comparing laparoscopic video trainers with simple box trainers.^11^ Based on a meta-analysis of five studies, they conclude that laparoscopic video trainers and simple box trainers are “equally proficient for the acquisition of laparoscopic skills”. Munshi et al. conducted a general review of HF versus LF simulators in clinical education and included two studies of procedural skills and conclude that HF is not always superior to LF and that the ideal simulator fidelity depends on the task being simulated.^12^ These results are similar to non-medically related simulation studies reported over 50 years ago.^9^

Another area of interest is the relationship of participant experience and simulator fidelity. Less experienced participants may gain significant educational benefit from lower fidelity simulators, while experienced participants may need higher fidelity simulators to realize an educational benefit. This idea was suggested by Alessi4 in studies from the aviation industry as well as in 1989 by Hays and Singer.^8^ This relationship has not been well studied in medical education to date.

It is timely to review the available evidence to guide the future development and use of future simulators, particularly in robotic surgery training. We undertook this literature review to evaluate the effect of simulator fidelity on training for procedural skills. This review focuses on the relationship between skills transfer and simulator fidelity for teaching procedural skills and the relationship of skills transfer and fidelity to the level of experience of study participants.

## Methods

A literature review methodology of existing studies was used. Only studies which conducted a direct comparison of HF and LF simulation for teaching a particular clinical procedural skill were included. There are no uniformly used criteria by which to classify simulators as HF or LF. Each author classified the simulators using no specific criteria and judged as LF or HF relatively within each study.

### Literature search strategy

**Figure 1:**
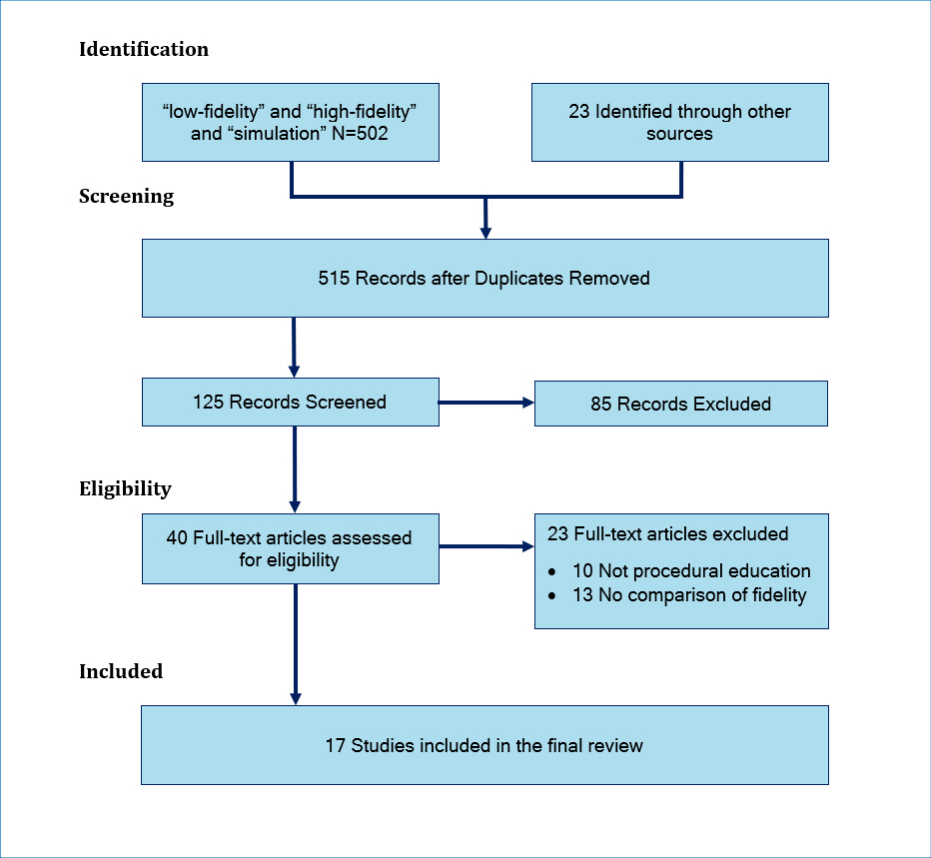
PRISMA chart showing the flow of studies in the review

A literature review was undertaken according to PRISMA guidelines^15^ to examine the effect of simulator fidelity on training in procedure skill training. MEDLINE, OVID and EMBASE databases were searched for articles on simulators that considered simulator fidelity published between January 1990 and January 2019. Search terms included “simulator fidelity and comparison” and "low fidelity" and "high fidelity" and “comparison” and “simulator”. Relevant articles from the search were identified by titles and abstracts. The full paper was then assessed. Reference lists from articles identified were also reviewed to identify additional studies not identified by the original search. The PRISMA flow chart is shown in Figure 1.

### Study selection and eligibility criteria

All included studies evaluate results based on simulator fidelity as the major outcome. Included studies use randomization to assign participants to LF or HF simulators. Studies with a didactic-only group, or a control group, or with a cross-over design are also included. Single group studies were excluded.

Studies which did not examine simulation of procedural skills were excluded, such as studies which simulated scenario management or learning physical examination. Only studies in the English language were included. Some studies provided extensive training in the simulation while others did not. Some studies included a didactic component for all participants while others did not. There were a variety of outcomes assessment tools used in the studies identified.

### Data extraction

The full text of the included studies was reviewed, and data extracted to a standardized data sheet (Excel, Microsoft, Redmond WA). The data extracted is shown in the headings of the Appendix. This instrument for critical appraisal was developed for the purpose of this study. Data abstracted included study design, nature of any interventions, study subjects, outcome measures (subjective, performance, clinical) and results (subjective, performance, clinical). The data abstracted were then reviewed.

### Quality appraisal

Data were abstracted by two investigators and the data sheets compared. Any discrepancies regarding conclusions were resolved by discussion. Discussions were held to yield a single document (Appendix) which summarized the 17 studies reviewed and in particular regarding the results of the study with regard to the effect of simulator fidelity on performance.

## Results

### Study selection

The result of the search for studies comparing performance on LF and HF simulators is shown in Figure 1. Studies which did not include a comparison of performance on HF and LF simulators were excluded. Finally,^17^ studies were reviewed in detail and included in this review.

The classification of fidelity used in these studies is complicated by the lack of a standard classification scheme. For example, in one study a video box was classified as LF^18^ while in another study the video box was classified as HF.^19^ Common categories of laparoscopic simulators are shown in Table 1, in increasing order of fidelity. We propose a classification scheme for laparoscopic simulator fidelity, to allow comparison of simulators labeled as Type 1 through Type 3 (Table 1). The simulators used in the studies reviewed were categorized by Type and then denoted as HF or LF in a consistent manner. All studies compared two types of simulators characterized as HF (higher type number in the classification in Table 1) and LF (lower type). For studies of non-laparoscopic simulator fidelity, we accepted the authors classification of HF and LF. This classification provides a standard description, including the simulation device itself and the constraints of the exercise.

### Study characteristics

Studies were reviewed, and data recorded in a standard format. Aggregate review data is shown in the Appendix. All studies included a randomization scheme for participants, and three studies included crossover.^19^^,^^20^^,^^21^ The simulation exercises in all studies were classified by simulator type, surgical constraints and task constraints. In 10 studies, there were two groups compared, with LF and HF simulators and five studies also had a didactic/control group.^22^^-^^26^ One study had a third group with progressive training using simulators with three levels of fidelity.^3^ One study compared three types of simulators.^27^

### Procedural skills

Of 17 studies reviewed, eight were focused on laparoscopic surgery skills. There were no studies evaluating robotic skills. The remaining nine studies evaluated simulation training for procedural skills, including trans-bronchial needle aspiration.20, vascular anastomosis,^28^ fiberoptic oral intubation,^29^ cricothyroidotomy,^30^ microvascular anastomosis,^24^ endourologic skills (basket stone removal),^22^ multiple emergency procedures,^23^ intravenous catheter placement^3^ and phlebotomy.^31^

### Evaluations

Of 17 studies reviewed, 13 evaluated participants one time and four studies evaluated participants both before and after training. One study used participant surveys only for evaluation.^20^ All other studies used a combination of global rating scores, time measurements, checklists and subjective evaluations by expert raters which included evaluation of recorded videos.

**Table 1:** Classification of laparoscopic surgery simulators

Simulator Type	Description
1	Simple box (e.g. plain cardboard box) with/without a mirror with a webcam or no video
2	Box trainer: box utilizing a camera/laparoscope display light source and instruments used for laparoscopic training (e.g. Fundamentals of Laparoscopic Surgery system)
2s	Box trainer (same as Type 2) with motion sensors to measure distance and direction moved in order to calculate the economy of movement
3	Virtual reality (VR) simulators: Display-based computer software and hardware similar to that used in laparoscopic surgery

### Simulator Fidelity and laparoscopic procedure skill training

This review includes eight studies designed to compare HF and LF simulators for laparoscopic surgery skill training. Simulators were classified on the scheme proposed here, Type 1 through Type 3. The classification of laparoscopy skill simulators is shown in Table 1. Five of eight studies compared Type 1 and Type 2 simulators. Five of eight studies compared two groups. Three of the studies had three groups (control or another simulator)^3^^,^^27^^,^^26^ and two studies had a crossover design.^19^^,^^21^

### Effect of simulator fidelity

Of nine studies of non-laparoscopic procedure training, 7/9 showed that training with the HF simulator was not better than training with a LF simulator. Review of laparoscopic surgery skill training studies showed that 8/8 concluded that training with an HF simulator did not have outcomes superior to training with LF simulators. Five studies included HF and LF groups as well as a didactic or control group.^22^^-^^26^ These five studies found similar outcomes with LF and HF simulators and that either LF or HF had better outcomes than a control group with no intervention or a group that received didactic training only.

The two studies that concluded that participants trained with HF simulators have better performance than those trained on LF simulators included a study of intravenous catheter insertion^3^ and a study of vascular anastomosis skills.^28^ In the vascular anastomosis study, time and global rating scores were similar for the LF and HF groups, but the HF group received a higher score on the overall performance in the animal surgery component of the study.

This review includes studies of a wide range of procedure skill training studies and shows that 15/17 studies have the similar conclusion, that training with an HF simulator does not have outcomes superior to training with an LF simulator.

### Effect of level of training

Of 17 studies reviewed, 14/17 used study participants at the same level of training so that no conclusions can be made regarding a relationship between fidelity, level of training and effect on performance. One study had participants at three levels (medical students, residents and faculty) but did not compare the effect of level of training on performance.^21^ One study included fourth year (USA) medical students and first-year residents and considered all participants in aggregate.^18^

One (1/17) study evaluated the performance of junior and senior surgery residents separately on HF and LF simulators.^28^ The authors concluded that skill acquisition was significantly affected by both simulator fidelity and level of training as measured by checklist scores and final product analysis on the animal surgery model. The global rating scores for junior and senior residents were similar for residents at both levels trained on both LF and HF simulators. The authors found that both junior and senior residents had better skill transfer from the bench model to the live animal model after practicing on HF simulators compared to LF simulators.

### Clinical outcomes evaluation

Of the 17 studies, 3/17 followed training with the evaluation of the skill on a patient (N=1) or a live animal surgery laboratory (N=2). The study with evaluation on a patient was for oral intubation in addition to laboratory performance on simulators, participants were evaluated by success rate, first-time success rate and time.^29^ Animal surgery included microsurgery^24^ and a vascular anastomosis.^28^ The microsurgery study evaluated final performance based on patency of the vas deferens. The vascular anastomosis study evaluated the quality of the vascular anastomosis based on an expert rater. Three studies followed training with the evaluation of a procedure on ex-vivo tissue.^20^^,^^23^^,^^26^

### Cost

The cost of the simulators used in the studies was stated in 4/17 studies. The LF simulator used for urology skills cost CDN $20, while the HF simulator cost CDN $3700.^22^ The cardboard box used for laparoscopic skills training cost €0, while the HF simulator was quoted as €30,000.^32^ Another laparoscopic skill study used three simulators including a simple webcam (USD $100), a mirror box (USD $300) and a video system (USD $2095).^27^ A laparoscopic skill study used an HF video trainer at the cost of $31,435 compared with a commercially produced camera-less box trainer which cost $185.^33^

## Discussion

This review of simulator fidelity and procedural skill training includes 17 studies of which eight were comparisons of simulator fidelity for teaching laparoscopic surgery skills. Overall, with some minor anomalies, the results of 15/17 studies reviewed are consistent in that while HF simulation and LF simulation are both effective for improving performance compared to no training or didactic training alone, HF simulators did not result in significantly improved performance compared to LF simulators. This was generally true across teaching a wide range of procedural skills. Two studies found improved performance with HF compared to LF simulation.^3^^,^^28^

The effect of simulator fidelity on performance has been studied for many years. Over 65 years ago, it was suggested that HF simulators do not result in improved skills transfer compared to LF simulators.^4^^,^^8^ These studies were not based on medical education but included data from a range of other fields such as aviation and manufacturing. Simulation and considerations of fidelity in medical education should be considered separately for examination skills, scenario management and procedural skills.

One of the problems in this type of comparison is a lack of a standard classification scheme. The LF simulator in one study is the HF simulator in another. Therefore, we propose a system for classifying simulation exercises to classify the simulator type (Table 1). All of the studies in the Appendix were classified in this manner to facilitate comparisons.

Data regarding the impact of simulator fidelity for scenario training is different than that for procedure training. In a study of performance in Advanced Cardiac Life Support, investigators concluded that those trained on HF simulators performed better.^34^   These results are not consistently reported, with a systematic review of scenario management simulation showing no difference in performance for participants trained on LF and HF simulators.^6^  In another scenario-based training simulation, 102 medical students were trained using LF or HF simulators and investigators that the students trained on HF simulators were over-confident and had worse performance than those trained on LF simulators.^35^

Data for procedure skill training does not generally show a benefit from training on HF simulators.  The availability of low-cost computing power has led to a range of HF simulators for laparoscopic training many of which are based on virtual reality (VR) technology. Despite their widespread use, VR trainers have been shown to be associated with improved skills transfer to the operating room only for basic skills and in laparoscopic cholecystectomy, and not for advanced procedures.^36^   In a systematic review of eight trials, six of which compared VR training to no training, VR was associated with improved operating time and performance, but an effect on clinical outcomes and cost has not been shown.^37^  In a meta-analysis of nine studies comparing VR trainers to box trainers, there was no significant difference in performance.^38^  Despite the fact that LF trainers can result in performance improvements similar to HF trainers, VR systems continue to be emphasized. A European consensus statement includes VR trainers as part of a proficiency-based training program.^39^

There have been several approaches to studying the effects of simulation training on participants at different experience levels. LF simulation in endovascular procedures was shown to improve motivation in novice trainees but does not necessarily improve practical skills.^40^ In one study of psychological stress, junior residents had significantly increased stress during HF simulation compared to LF simulation, while senior surgeons showed no differences.^41^ It was also suggested that HF simulators might result in poorer performance by novice trainees because they are over-stimulated by the HF environment.^9^ A meta-analysis of VR trainers was not able to conclude about the effect of level of training on effectiveness of VR simulators compared to box trainers.^38^ In one study reviewed here, medical students with no prior experience performed better with a LF simulator than a HF simulator.^22^ Existing data are insufficient to draw a conclusion about level of experience and simulator fidelity. In a single study, investigators compared junior and senior resident performance separately and found improved skill transfer after using a HF simulator for vascular surgery.^28^ In a systematic review, investigators compared the performance of laparoscopically naïve participants after training with a Type 1 LF simulator (box trainer) and found their performance better than those who had no training.^42^ The effect of level of training on the benefits derived from simulators of different fidelity needs further study.

Simulators classified as HF are generally more expensive than LF simulators. Using the most appropriate simulator for the target audience is important for efficient use of resources. In a systematic review of low-cost simulators, investigators reviewed 73 unique simulators which cost <£1500, including 60 non-commercial and 13 commercially available simulators.^14^ They reported that commercial simulators ranged from £60 to £1007 and non-commercial simulators ranged from £3 to £216. Of the 17 studies reviewed here, the cost was mentioned in just three studies. The LF endourology model cost CDN $20 and the HF model cost CDN $3700.^22^ In a study of three laparoscopic simulators, the Type 2 HF video system cost USD $2095, the Type 1 mirror box cost USD $300 and the Type 1 webcam system was USD $100.^23^ In another study of laparoscopic trainers, the conventional Type 2 video system cost €30,000, while a no-cost Type 1 cardboard box provided equal training benefits. The general lack of cost information reporting has been noted in other reviews.^38^ These results stress the importance of reporting costs in future studies of simulator training to allow comparisons of this important parameter.

The ultimate goal of simulation training is to improve clinical outcomes for patients. This remains difficult to prove. In this review, 3/17 studies followed simulation training with a patient intervention or a live animal laboratory. In a study of VR training versus a control group with a total of 13 residents, trainees who completed VR training had improved results in their first ten laparoscopic cholecystectomy procedures (fewer errors, faster times) compared to the control group.^43^ Future studies should include clinical exercises after simulation training when possible.

The results of this literature review have implications for procedure skill training in the future. It is evident from numerous studies that training with a simulator (HF or LF) results in improved performance compared to no training at all or didactic training alone.^22^^-^^26^^,^^42^ All but two of the 17 studies reviewed did not find that HF simulators (also generally more expensive than LF simulators) result in the improved acquisition of procedural skills compared to LF simulators. This could significantly affect budgets for simulation education since it may not be necessary to always use a HF simulator.

Few studies have examined the effect of participant training level on simulation training. It may be necessary to use HF simulators to train people with more experience while LF simulators may be perfectly adequate for those with little or no experience. The paucity of data in the literature makes it difficult to draw conclusions and supports future studies of this issue which also has implications for the cost of training. More studies are needed with participants having varied levels of experience to determine who will benefit most from different types of simulators. The need to provide objective feedback to trainees is emphasized by a number of studies and should be part of future studies.  Simulator fidelity is another parameter to be considered in the design of simulation curricula as well as future trials.

Training in robotic surgery is emphasized in contrast to what happened when laparoscopic surgery was introduced.^2^ Simulators designed for robotic-assisted surgery training are generally high-fidelity, large and expensive because they closely resemble the actual daVinci robotic-assisted surgical system robot (Intuitive, Sunnyvale CA) which is the most commonly used system. A reasonably low-cost portable VR trainer for the daVinci system was recently described.^44^ The Fundamentals of Robotic Surgery course has been developed and validated and is becoming more widespread.^45^ The FIRST exercises have also been validated.^46^  However, a meta-analysis of 107 studies concluded that there is no universally accepted method of robotic surgery skill assessment.^47^  In a systematic review of robot-assisted surgery VR simulators, investigators concluded that it is not clear which exercises and metrics can distinguish levels of training in performance on the daVinci system.^48^  Further study is essential as training in robot-assisted surgery becomes more widespread.

There are acknowledged limitations of this review. First, there is some heterogeneity of the included studies, particularly regarding methods of evaluation. The included studies cover a wide range of procedural skill training using various methods for evaluation of outcomes. Comparisons of studies are somewhat limited by lack of a standard scheme for classification of simulators, and comparisons in the included studies are based on relative fidelity. There is a wide range of simulators compared, which makes comparisons difficult. In addition, the training schemes used are widely variable. Future studies would benefit from a unified scoring system for the assessment of outcomes.

## Conclusions

Despite some heterogeneity of the 17 included studies, there are several important conclusions from this review. Of the studies evaluated, 15/17 show that LF simulation results in similar outcomes to HF simulation. LF simulators may provide significant educational benefits for less experienced trainees while HF simulators may be of benefit to more experienced trainees. These results may lead to cost savings in medical education since simulators that are generally less expensive provide similar training to more expensive simulators. We suggest that future studies of laparoscopic simulation education adopt the classification scheme introduced here to facilitate future comparisons. A lack of uniform criteria for evaluating procedural skills remains a formidable barrier to determining whether or not simulation training results in improved clinical performance, which is the ultimate goal of simulation education and requires further study.

### Acknowledgements

The authors gratefully acknowledge the contributions of Murilo Marinho PhD to this project.

### Conflicts of Interest

The authors declare that they have no conflict of interest.

## Supplementary material

Supplementary file 1 Appendix. Seventeen studies of the effect of simulator fidelity on procedure skill training
