# Supplementary material for: The effect of simulator fidelity on procedure skill training: a literature review
Source: Int J Med Educ. 2020 May 18;11:97–106. doi: 10.5116/ijme.5ea6.ae73 (PMC7246118; doi:10.5116/ijme.5ea6.ae73)
Supplement: Supplementary file 1 — Appendix. Seventeen studies of the effect of simulator fidelity on procedure skill training [file ijme-11-97-S1.pdf]

Appendix

Seventeen studies of the effect of simulator fidelity on procedure skill training

Ref	Year	Procedural Skill	Study Design / Intervention / Exercise Classification	Study Subjects	Outcome Measures/Evaluations	Clinical Evaluation*	Results: Opinion, Performance, Clinical
**Non-Laparoscopic Skills**							
20	2009	Trans-bronchial needle aspiration	-2 groups, Randomized Crossover: LF (rubber tube) and HF (VR system)<br>-All participants trained on both LF and HF	44 practicing physicians	-Opinion: Participant Likert-scale surveys completed after training on both types (LF/HF) and then a survey after completing both<br>-Performance: Not evaluated<br>-Clinical: Not evaluated	No	LF preferred<br>-Opinion: LF preferred 19/44 vs HF 11/44. LF was judged more realistic 23/44 vs 17/44 for HF<br>-Performance: Not evaluated<br>-Clinical: Not evaluated
3	2010	IV insertion	-Randomized to LF (Virtual IV, Laerdal), Mid-fidelity (plastic arm), HF (Sim-Man, Laerdal) or progressive practice.<br>-After practice on LF or HF, or with progressive from LF to medium to HF, tested once on a standard simulator	45 medical students	-Opinion: Not evaluated<br>-Performance: Blinded raters scored Integrated Procedural Performance Instrument Rating, Global Rating Scale, Checklist and Communications<br>-Clinical: Not evaluated	No	Progressive from LF-Medium-HF is best, HF better than LF<br>-Opinion: Not evaluated<br>-Performance: Progressive from LF to medium fidelity to HF better than HF (effect size 0.78); HF better than LF (effect size 0.72). Progressive group had more practice time overall, but less on the HF<br>-Clinical: Not evaluated
29	2008	Fiberoptic oral Intubation	-Randomized to LF (bronchoscope, simple bench model) or HF (VR, Accutouch Flex bronchoscopy simulator) simulators followed by<br>-Clinical skill test on patient	28 respiratory therapists	-Opinion: Not evaluated<br>-Performance: Objective checklist, time, success rate and GRS<br>-Clinical: GRS, Success rate, time, first-attempt rate	Yes	LF=HF<br>-Opinion: Not evaluated<br>-Performance: GRS, checklist, LF=HF<br>-Clinical: GRS, checklist scores same LF=HF, success rates same, both LF and HF showed equal improvement first to second attempts
22	2002	Endourology-stone removal	-Randomized 3 arm: didactic, LF (self-designed) and HF (VR Model, Limbs & Things, Bristol UK)<br>-One evaluation after LF/HF or didactic.<br>-LF model CDN $20, HF model CDN $3700	40 medical students	-Opinion: Not evaluated<br>-Performance: GRS, checklist and time<br>-Clinical: Not evaluated	No	LF=HF, HF and LF both superior to didactic<br>-Opinion: Not evaluated<br>-Performance: GRS LF 79% vs. HF 88% (p=0.08), Checklist LF 90% vs. HF 94% (p=0.17), overall pass rating p=0.95<br>-Clinical: Not evaluated

23	1999	Six Procedures: burr hole, chest tube, bowel anastomosis, wound closure, tendon repair, K-wire insertion	-Randomized 3 arms: didactic, HF (cadaver), or LF (dry model)<br>-One-time evaluation on cadaver model of each group for all 6 procedures	23 residents	-Opinion: Not evaluated<br>-Performance: GRS and checklist<br>-Clinical: Not evaluated	No	LF=HF, HF and LF both superior to didactic<br>-Opinion: Not evaluated<br>-Performance: GRS LF 64%, HF 67%, p>.05; Checklist LF 68%, HF 69%, p>.05<br>-Clinical: Not evaluated
24	2004	Microsurgery	-Randomized to 3 groups for training: didactic, LF (silicone tube) and HF (rat vas deferens).<br>-All participants evaluated on both LF and HF<br>-Animal surgery by all participants. One-time evaluation	50 residents	-Opinion: Not evaluated<br>-Performance: GRS, checklist, time<br>-Clinical: patency of vas deferens anastomosis in a rat model	Yes	LF=HF, HF and LF both superior to didactic<br>-Opinion: Not evaluated<br>-Performance: GRS LF 64%, HF 70%, checklist LF 84%, HF 89%, p>.05<br>-Clinical: Anastomotic patency same LF=HF groups, both better than didactic
28	2007	Vascular Anastomosis	-Randomized to HF (cadaver) or LF (plastic model)<br>3hr simulator training either LF or HF.<br>-Animal surgery after training<br>-One-time evaluation after animal surgery. Analyzed junior and senior residents separately	27 Surgery residents	-Opinion: Not evaluated<br>-Simulator Performance: Not evaluated<br>-Clinical: Checklist, GRS, time, hand motion, final product	Yes	GRS LF=HF for Junior and Senior residents. HF final product better than LF for Junior and Senior residents.<br>-Opinion: Not evaluated<br>-Simulator Performance: Not evaluated<br>-Clinical: Final product analysis: Junior LF (3.1), HF (4.0), p<.05, Senior LF (3.6), HF (5.0), p<.05. GRS scores LF=HF both junior and senior
30	2018	Cricothyroidotomy	-Randomized to HF (3D printed larynx) or LF (simple tube)<br>-Lecture for all then training on LF or HF, 10 times then *ex-vivo* -porcine larynx	52 residents	-Opinion: similarity of simulator to porcine larynx<br>-Performance: success rate, time using porcine larynx<br>-Clinical: Not evaluated	No	LF=HF for opinion and performance<br>-Opinion: Both groups reported similar appearance to porcine larynx (LF 3/5, HF 4/5, p=0.81).<br>-Performance: HF and LF no difference. Success rate (LF 52%, HF 48%, p>.05), time (45 vs 49sec, p>.05)<br>-Clinical: Not evaluated
31	2006	Phlebotomy	-Randomized to HF (VR) or LF (simulated arm)<br>-Before/After evaluation	45 third year medical students	-Opinion: Not evaluated<br>-Performance: Checklist, pretest, post-test. Compared before/after scores for each training method<br>-Clinical: Not evaluated	No	LF scores higher than HF<br>-Opinion: Not evaluated<br>-Performance: Post-test scores higher for LF compared to HF<br>-Clinical: Not evaluated

**Laparoscopic Skills**							
24	2004	Laparoscopic skills- cutting and clip application	-Randomized to 3 groups: control, HF (Type 3, box, cutting), -LF (Type 2s, box, cutting).<br>Before/ after evaluation	24 junior residents	-Opinion: Not evaluated<br>-Performance: Motion data (both LF and HF), time on pre-test, then training (3 groups), then reassessment<br>-Clinical: Not evaluated	No	LF=HF, HF and LF both superior to controls<br>-Opinion: Not evaluated<br>-Performance: Motion, time LF=HF, both LF and HF better than control<br>-Clinical: Not evaluated
18	2011	Five laparoscopic skills: camera handling, knot tying, scope tracking, clipping, needle transfer	-Randomized to HF (Type 3, box, pegs/suture/cutting) simulator or LF (Type 2, box, pegs/suture/cutting).<br>-6-months of training<br>-Evaluation on porcine model (live tissue) at 0,2,6 months	11 PGY-1 residents and 12 fourth-year medical students	-Opinion: Not evaluated<br>-Performance: checklist, video review, porcine surgery to test the skills<br>-Clinical: Not evaluated	Yes	LF=HF, students and residents improved<br>-Opinion: Not evaluated<br>-Performance: Not evaluated on simulators<br>-Clinical: Porcine model, both LF and HF groups showed improved scores in all 5 skills from 0 to 2 to 6 months, LF vs HF p>.05
19	1999	Seven basic laparoscopic skills	-Randomized to LF (Type 1, box, pegs/suture/cutting)) or HF (Type 2, box, pegs/suture/cutting)<br>-Crossover<br>-One-time evaluation on both LF and HF	22 surgery residents	-Opinion: Not evaluated<br>-Performance: Precision and speed scores<br>-Clinical: Not evaluated	No	LF (1,499 ± 237) = HF (1,209 ± 435, p>.05)<br>-Opinion: Not evaluated<br>-Performance: Scores correlated with level of training and the mirrored box scores differentiate junior and senior residents<br>-Clinical: Not evaluated
33	2005	Four basic laparoscopic skills	-Pre-test on HF (Type 2, box, blocks/suture/cut)<br>-Then randomized to 5 hours training on LF (Type 1, box, blocks/suture/cut) or HF (Type 2, box, blocks/suture/cut)<br>-Post-test on HF simulator	22 Urology residents	-Opinion: asked if simple trainer was effective at post-test<br>-Performance: GRS and time/efficiency<br>-Clinical: Not evaluated	No	LF=HF for improvement of scores at post-test<br>-Opinion: 91% felt that the simple trainer was effective<br>-Performance: All participants improved at post-test compared to pre-test, p<.05. GRS for LF (43,.91) and HF (55.87, p>.05). Time and efficiency for all 4 skills improved by the same amount for LF and HF.<br>-Clinical: Not evaluated
27	2005	Peg transfer and pattern cutting	-Randomized to 3 groups<br>-Compared 3 simulators: LF (Type 1, box, peg/cutting compared to a commercial LF (Type 1, (box, peg/cutting) and HF (Type 2, box, peg/cutting)<br>-Six groups tested on all 3 simulators used in a different order	42 laparoscopically naïve residents	-Opinion: Not evaluated<br>-Performance: Time and performance scores<br>-Clinical: Not evaluated	No	LF (webcam) same as HF. Either of these better than LF (2-mirror)<br>-Opinion: Not evaluated<br>-Performance: Time compared, and order of simulators did not make a difference<br>-Clinical: Not evaluated

26	2007	Laparoscopic suturing	-Randomized to 3 groups: control, LF (Type 1, box, knot) and HF (Type 2, box, knot)<br>-Video review of all suturing<br>-Final evaluation on cadaveric animal model	30 medical students	-Opinion: Not evaluated<br>-Performance: time for suturing and review of videos<br>-Clinical: Not evaluated	No	LF=HF, either is better than control<br>-Opinion: Not evaluated<br>-Performance: mean times: control (12min), LF (6.6min), HF (6.6min)<br>-Clinical: Not evaluated
32	2006	Laparoscopic Skills: Sugar cube transfer, mint transfer and disk cut-out	-Randomized to 24h (8 sessions) training on either HF (Type 2, box, pegs/cutting) or LF (Type 1, box, pegs/cutting)<br>-Evaluated after training one time on both simulators	36 medical students	One-time test on both simulators<br>-Opinion: Not evaluated<br>-Performance: Accuracy, time, depth perception assessed subjectively.<br>-Clinical: Not evaluated	No	LF=HF for all tasks tested<br>-Opinion: Not evaluated<br>-Performance: Subjective scores for all tasks by blinded rater were similar for all 3 tasks for both groups ($p>.05$). Times were similar ($p>.05$). LF group had better accuracy score on disk cut-out than HF. LF trained participants were faster when tested on the HF simulator.<br>-Clinical: Not evaluated
21	2012	Laparoscopic skills: skill training was different for LF and HF	-Randomized with crossover: LF (Type 2, box, pegs/cutting/suturing) and HF (Type 3, box, pegs/cutting/suturing)<br>-After achieving minimal proficiency on LF or HF, crossed over to the other simulator and tested	228 medical students, residents and senior physicians	-Opinion: Not evaluated<br>-Performance: Scores on FLS box and LapSim VR. Baseline scores compared with scores after training on the other simulator<br>-Clinical: Not evaluated	No	LF-trained participants did better on HF than HF-trained participants did on LF<br>-Opinion: Not evaluated<br>-Performance: LapSim trained participants had a 20% task pass rate while FLS to LapSim trained participants had a 29% pass rate ($p<.01$). Score increase similar ($p>.05$) after training on either device.<br>-Clinical: Not evaluated

LF: Low fidelity; HF: High fidelity; FLS: Fundamentals of Laparoscopic Surgery; LapVR: CAE Healthcare (Sarasota FL USA); LapSim: Surgical Science (Gotheborg, Sweden); VR: Virtual Reality
*Clinical evaluation indicates that skill was tested on a patient or live animal after training; GRS: Global Rating Scale
